# Surveillance of FAP: a prospective blinded comparison of capsule endoscopy and other GI imaging to detect small bowel polyps

**DOI:** 10.1186/1897-4287-8-3

**Published:** 2010-04-04

**Authors:** Paul Tescher, Finlay A Macrae, Tony Speer, Damien Stella, Robert Gibson, Jason A Tye-Din, Geeta Srivatsa, Ian T Jones, Kaye Marion

**Affiliations:** 1 Department of Medicine, The University of Melbourne, Melbourne, Australia; 2 Department of Colorectal Medicine and Genetics, The Royal Melbourne Hospital, Melbourne, Australia; 3 Department of Radiology, The Royal Melbourne Hospital, Melbourne, Australia; 4 Department of Mathetmatics and Statistics, Royal Melbourne Institute of Technology, Melbourne, Australia

## Abstract

**Background:**

Familial adenomatous polyposis (FAP) is a hereditary disorder characterized by polyposis along the gastrointestinal tract. Information on adenoma status below the duodenum has previously been restricted due to its inaccessibility in vivo. Capsule Endoscopy (CE) may provide a useful adjunct in screening for polyposis in the small bowel in FAP patients. This study aims to evaluate the effectiveness of CE in the assessment of patients with FAP, compared to other imaging modalities for the detection of small bowel polyps.

**Method:**

20 consecutive patients with previously diagnosed FAP and duodenal polyps, presenting for routine surveillance of polyps at The Royal Melbourne Hospital were recruited. Each fasted patient initially underwent a magnetic resonance image (MRI) of the abdomen, and a barium small bowel follow-through study. Capsule Endoscopy was performed four weeks later on the fasted patient. An upper gastrointestinal side-viewing endoscopy was done one (1) to two (2) weeks after this. Endoscopists and investigators were blinded to results of other investigations and patient history.

**Results:**

Within the stomach, upper gastrointestinal endoscopy found more polyps than other forms of imaging. SBFT and MRI generally performed poorly, identifying fewer polyps than both upper gastrointestinal and capsule endoscopy. CE was the only form of imaging that identified polyps in all segments of the small bowel as well as the only form of imaging able to provide multiple findings outside the stomach/duodenum.

**Conclusion:**

CE provides important information on possible polyp development distal to the duodenum, which may lead to surgical intervention. The place of CE as an adjunct in surveillance of FAP for a specific subset needs consideration and confirmation in replication studies.

**Trial Registration:**

Australian New Zealand Clinical Trials Registry ACTRN12608000616370

## Background

Polyps occur in the upper gastrointestinal tract in 30-100% of patients with Familial Adenomatous Polyposis [1,2]. Duodenal polyps (present in 45-90% of patients) are usually adenomatous and have a 4-12% cancer risk. Adenomas in the distal small bowel and in the stomach have a lower cancer risk than duodenal adenomas, although cancer in an ileostomy following colectomy can occur [3-5]. The number of patients dying with adenocarcinoma of unknown primary is not well documented in the literature and could include patients developing small-bowel cancer through an adenoma-cancer pathway [6].

Screening of the upper gastrointestinal (GI) tract with side-viewing (SV) endoscopy for gastric and duodenal polyps has been recommended [7,8]. Other current techniques, including small-bowel follow through (SBFT) and MRI have had limited evaluation in the setting of FAP.

Since its advent in 2000 [9], capsule endoscopy (CE) has proved useful for the investigation of the small bowel [10,11]. CE offers potential advantages over other forms of imaging, in terms of diagnostic sensitivity and specificity, safety and tolerability [12,13]. Unlike most other endoscopic techniques, CE allows visualisation of the entire small bowel, including jejunum and ileum.

Further exploration of the utility of CE may help to identify if it is a useful adjunct to standard endoscopy as a screening protocol in FAP. This could lead to more accurate and earlier diagnosis of possible adenomatous polyps, particularly if located in the jejunum and ileum, potentially resulting in decreased morbidity and mortality rates for FAP patients.

The aim of this prospective blinded study was to establish CE's sensitivity and specificity for detection of polyps in each part of the small bowel, compared with other imaging modalities, in patients with FAP.

## Methods

Twenty consecutive patients with previously diagnosed FAP and duodenal polyps, presenting for routine duodenal surveillance at The Royal Melbourne Hospital were recruited. Patients were considered eligible if they had previously diagnosed FAP with duodenal polyps and were over the age of 18 years old. Patients also must have had clinical or X-ray assessments to assess potential obstruction of the small bowel before being considered for this study.

Exclusion criteria were suspected stenosis or obstruction of the small bowel, pregnancy, a swallowing disorder that would preclude the safe ingestion of the capsule, cardiac pacemaker or other implanted electromedical devices. If a patient was expected to undergo MRI examination before the elimination of the capsule, they were excluded as MRI scanning can potentially interfere with transmission signals and may disrupt the integrity of the capsule. The study protocol was approved by the Melbourne Health Human Research Ethics Committee and informed consent was obtained from each patient.

Each patient underwent four investigations to image the bowel within a six week timeframe. Initially the patient underwent a MRI of the abdomen. The MRI was performed on the fasted patient and scanned using 1 litre of dilute barium as intra luminal contrast. Scanning was performed with 5/1 mm thick T2SSFSE and pre/post T1 fat-saturated GRE breath-hold sequences in the axial and coronal planes. One week later, a barium SBFT study was performed to exclude small bowel obstruction and as an assessment of small bowel polyposis.

Providing that there was no evidence of small bowel obstruction, the patient proceeded to CE ("Pillcam M2A" Capsule, Given Imaging, Yoqneam, Israel), four weeks later. The technical description is available elsewhere [9]. Each patient was required to fast at least 8 hours prior to capsule ingestion, with no additional bowel preparation given directly prior to their CE and to remain within the hospital for the 8 hour duration of the CE transmission period.

An upper gastrointestinal endoscopy using a side-viewing scope was done one to two weeks after CE, with the procedure recorded on digital video. Biopsies were taken of polyps >5 mm as per routine practice in surveillance. Duodenal and small bowel adenomas detected in the course of the study were managed outside the protocol, on clinical merits including with polypectomy or ablative therapy.

Endoscopic examinations were performed by one of two experienced endoscopists. For all four procedures, the imaging was read by two investigators with specialty training and expertise in their procedure (gastroenterologists for CE and endoscopy, radiologists for SBFT and MRI). One investigator was blinded to the patient history and results of all investigations performed on the patient. The second investigator was not blinded and read all studies in the series to ensure consistency. Where there was a discrepancy between findings by the two investigators, an independent blinded assessor reviewed the images to decide the findings.

Localisation on CE was determined using known observable anatomic features of the bowel, including identification of the major and minor ampullae, pylorus and ileo-caecal valve. Localization was also judged by analyzing the time scale between passage through the pylorus and the ileo-caecal valve, informed by the localization software provided by Given Imaging.

Pathologic findings were recorded for each imaging modality. The estimated polyp size and number of polyps in each segment of the gastrointestinal tract (gastric, duodenum, jejunum, ileum, caecum) was documented. Numbers of polyps were recorded as 1, 1-4, 5-20 and >20. Size estimation of polyps was recorded as 1-5 mm (small), 6-10 mm (medium) and >10 mm (large) judged with reference to open biopsies forceps as calibration (standard endoscopy) or with reference to small bowel morphology. The severity of duodenal polyposis, classified using a modified version of the Spigelman classification system was noted, but not used [14].

Student's t-test/chi square testing were used for a comparison of polyp numbers and size, and identification of abnormal papillae, as estimated by each method. Analysis of variance was conducted using a General Linear Model and Two-Way ANOVA. Within the duodenum sensitivity and specificity of the Capsule was calculated using the duodenoscopy findings as the "gold standard".

## Results

A total of 20 patients were investigated in this study. The average age was 44.7 years (range 19-79) with equal numbers of males and females.

**Figure 1:**
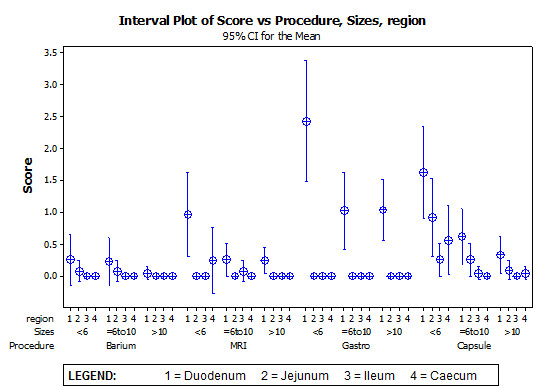
**Interval Plot of Score vs Procedure, Sizes, Region**.

A square root calculation was performed on the total polyp numbers to equalize the variance of the observations (valid for the Poisson distribution of this dataset).(Figure 1) Two-way ANOVA calculation to compare each modality and region gave p-value < 0.001.

Overall, upper SV endoscopy provided the most findings in the segments of stomach and duodenum, and CE provided most findings beyond the duodenum. In the duodenum, upper SV endoscopy identified more total polyps than the other three procedures for 13 patients and either the same or fewer than CE procedure for 6 patients(p < 0.001). For one patient, no polyps were detected with either upper GI SV endoscopy or CE, whereas SBFT and MRI both identified 5-20 small polyps for this patient.

When comparing the total number of polyps per size, upper GISV endoscopy identified more polyps >10 mm than other modalities, fewer polyps <6 mm than CE and was similar to CE for polyps 6-10 mm.

Few polyps were diagnosed overall by the barium follow through or MRI, with CE identifying more polyps in regions other than the duodenum. For all regions the majority of polyps diagnosed by CE were size <6 mm.

SBFT produced the fewest findings of all investigations, detecting polyps in 8 of 20 (40%) patients. SBFT detected stomach polyps in 4 patients, but none greater than 5 mm were identified. Five patients were diagnosed with duodenal polyps (2 with polyps <5 mm, 2 with polyps 6-10 mm and 1 patient with a polyp >10 mm). Only 1 patient provided findings on SBFT beyond the duodenum, with 1-4 polyps up to 10 mm in the jejunum.

MRI studies revealed polyps in 11 of 20 (55%) patients. These polyps were predominantly localized to the duodenum, although there was at least one finding for each of stomach, ileum and caecum. MRI was able to detect a polyp larger than 10 mm in 5 out of the 11 patients with polyps of this size; of these, none of the polyps were located outside the duodenum. In comparison to CE, MRI detected fewer polyps across all parts of the bowel. It also detected fewer polyps of all sizes than CE.

Upper GI SV endoscopy was able to identify at least one finding in 19 of 20 (95%) patients, detecting gastric polyps in 17 of 20 (85%) and duodenal polyps in 18 of 20 (90%) of patients. The predominant finding was >20 small (1-5 mm) polyps. No polyps were identified by upper gastrointestinal SV endoscopy beyond the duodenum.

The average gastric emptying time measured by the capsule was 38 minutes (range 3-149 minutes) and the average small bowel transit time was 4 hours 15 minutes (range 2.13-7.25 hours). In 3 patients a total small bowel transit time could not be accurately determined. One of the capsule studies reported views indicative of the large bowel, but could not clearly identify the point where the capsule reached the ileo-caecal valve. In another patient, the capsule remained in the stomach for the duration of the study.

**Figure 2:**
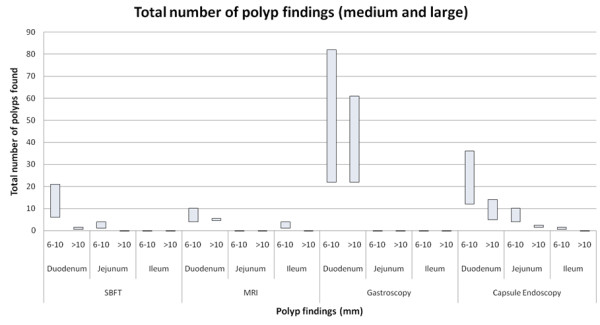
**Total number of polyp findings (medium and large)**.

CE was the only form of imaging in this study that identified polyps in all segments of the bowel. It provided a significantly higher total number of polyp findings in jejunum, ileum and caecum than MRI and SBFT (Figure 2).

CE produced significantly fewer findings than SV endoscopy in stomach and duodenum. This was primarily attributed to CE significantly underestimating number of polyps in 9 studies. Six of these 9 (66%) underreported the number of duodenal small (1-5 mm) polyps, 2 of 9 (22%) underestimated medium polyps (6-10 mm) and one CE reported a fewer number of >10 mm polyps than for the same patient on upper gastrointestinal endoscopy.

Interestingly, CE detected a number of polyps that were not identified by upper gastrointestinal endoscopy in some studies. There were 3 findings by CE in the stomach not found on upper gastrointestinal endoscopy (including 5-20 small polyps and 1 medium polyp). In the duodenum, CE detected 3 instances of polyps not found on SV upper gastrointestinal endoscopy (up to 20 small polyps, 1-4 medium and 1-4 large polyps).

**Figure 3:**
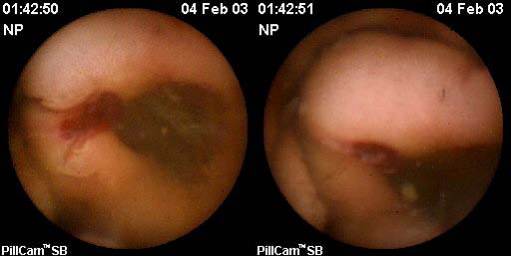
**Images taken by CE of a 25 mm bleeding tumour in the jejunum**.

In one patient, CE detected a 25 mm bleeding polyp at the duodeno-jejunal flexure (Figure 3). The polyp was histologically identified as an advanced adenoma following surgical resection. This finding was missed on standard endoscopy. This case indicates that clinically significant polyps may be missed with standard SV endoscopic surveillance and CE may be of benefit as an adjunctive screening test.

## Discussion

Patients with Familial Adenomatous Polyposis have a greatly increased lifetime risk of developing adenomas and carcinomas in the colon, stomach and duodenum [14-16]. These dysplastic lesions may be associated with significant complications including metastases and bowel obstruction [17-19]. Treatment of FAP patients can often be difficult due to the widespread nature of their polyposis. Total colectomy is recommended for established large bowel polyposis, but this does not reduce the risk of adenomatous growths along other parts of the bowel. Likewise resection of the duodenum has been found only moderately effective as a treatment strategy as neoplastic polyps may still develop within jejunum or ileum after the Whipple's resection.

The current recommended guideline for treatment includes regular screening for the detection and monitoring of polyps within FAP patients, with colectomy advised in a timely fashion after establishment of the diagnosis. SV upper gastrointestinal endoscopy is highly effective for identifying most polyps within the duodenum. However the possibility of adenomas developing in segments of the bowel inaccessible by standard upper GI endoscopy in a proportion of FAP patients indicates that additional modes of screening could be considered.

Based on our results. we suggest that both MRI and SBFT detect significantly fewer gastric and duodenal polyps compared to upper gastrointestinal SV endoscopy or CE. Beyond reach of duodenoscopy, however, our data suggests that MRI and SBFT also detect significantly fewer polyps than CE. The low frequency of findings on these two forms of imaging support the previous research which indicate MRI and SBFT are unsuitable for primary screening for polyps in FAP [20-22].

The decreased sensitivity of CE for gastric and medium to large duodenal polyps conforms with previous experience [23], with a consensus opinion that CE has limited ability to detect lesions proximal to the ampulla due to the relatively short transit time by the capsule through the first part of duodenum. Fast gastric emptying times and the poor ability to localize the capsule within the stomach could help explain the underestimation of gastric polyps. In light of the high incidence of polyps around the papilla of Vater, we recommend that upper gastrointestinal endoscopy should remain the primary form of screening for duodenal polyps.

Previous research has indicated that CE may have a significant role in the detection of small bowel pathology including polyps and cancer [24,25]. Schulmann et al demonstrated the ability of CE to detect a high frequency of polyps in the distal bowel of FAP patients [15]. Our data supports these conclusions, with a significant range of findings, including medium to large polyps identified within the jejunum and ileum by CE. However in this study we were unable to confirm distal polyps with biopsy and it is possible that some abnormalities identified as polyps may not be adenomatous and could be other possibilities, for example lymphoid hyperplasia.

CE is also limited by its inability to objectively determine polyp size due to lack of standard reference. This may result in the underestimation of polyp size by CE. There may be a role for the use of ingested standardized markers [26]. Further investigation of the utility of these markers in assessing polyp sizes may help establish their suitability for standard CE protocol.

Published literature has largely ignored the prevalence of cancer in the jejunum and ileum of FAP patients, mainly due to the limited accessibility of this region to upper GI SV endoscopy. One Danish study examining all causes of death in a FAP population indicated there may be a small risk of jejunal cancer developing in this population [6]. Two other studies found a high correlation between the Spigelmen Score and more distal polyps found on CE [15,27]. Based on these and our findings we would suggest that CE be considered for further screening in selected patients with FAP.

Clinically, cancers in the small bowel distal to the duodenum have not been considered common in FAP. In so far as adenomas are precursors to cancer, our findings challenge the usual clinical surveillance strategies of focusing only on the duodenum and colon, because of the apparent low cancer risk in jejunum and ileum. Since the formulation of the current standard for FAP surveillance, there have been multiple developments which may necessitate a change in standard practice.

These developments include the increasingly effective cancer control in the duodenum and colon leading to increased survival of FAP patients (through polypectomy or surgery), an uncertain incidence of deaths from disseminated adenocarcinoma (?primary in small bowel) and greater facility to endoscopically remove polyps in the jejunum and ileum through the advent of double-balloon enteroscopy. Double-balloon enteroscopy was not used as an intervention in this study as it was not available at the time, but it may provide useful information to help confirm CE findings in FAP surveillance. Although double-balloon enteroscopy has the advantages of biopsy and interventional capabilities, CE is a less invasive procedure and is well tolerated.

Further confirmatory studies might help evaluate the significance of CE, by assessing the clinical correlation between distal polyps detected by CE and the subsequent health outcomes. The comparison of newer endoscopic techniques, such as double-balloon enteroscopy with CE might be useful in further defining the sensitivity and specificity of CE for distal polyps. Our study has shown CE to be of limited effectiveness for assessment of gastric and duodenal polyposis, but frequently identifies distal bowel polyps and is likely to be clinically relevant for a subset of patients at higher risk of distal small bowel adenomas. These results affirm the findings of previous studies [21], and suggests that CE may play an important role within a FAP screening protocol.

## Conclusions

Side-viewing upper GI endoscopy and capsule endoscopy were able to detect a significantly higher number of polyps in the bowel than MRI and small bowel follow through. Upper GI endoscopy excelled at identifying polyps in the stomach and duodenum, but a significant proportion of findings were detected by capsules that were not seen on standard endoscopy. The low sensitivity of CE in detecting large duodenal polyps of greatest clinical relevance indicates that CE would be unsuitable as a primary screening tool for FAP patients. Capsule Endoscopy identifies a greater number of bowel polyps distal to the duodenum in a subset of patients with FAP.

Capsule Endoscopy may be useful as an adjunct in FAP screening and is likely to be clinically relevant for a subset of patients at higher risk of distal small bowel adenomas.

## List of Abbreviations

FAP: Familial Adenomatous Polyposis; MRI: Magnetic Resonance Imaging; SBFT: Small-Bowel Follow Through; CE: Capsule Endoscopy; GI: Gastrointestinal; SV: Side-viewing.

## Competing interests

The authors declare that the capsules for this study were provided by Given Imaging, but the study was entirely investigated, initiated, and analysed independently by our colleague at the Royal Melbourne Institute of Technology Statistics Department (KM). The PI has been a regular member of teaching faculties run by Given Imaging in Australia and elsewhere.

## Authors' contributions

FA was responsible for conception & design of this study. TS, DS, RG, JT, GS and IJ were responsible for data acquisition. KM performed the statistical analysis and interpretation of the data. PT was responsible for drafting and revising the manuscript. All authors read and approved the manuscript.
